# Black Poplar (*Populus nigra* L.) Root Extracellular Trap, Structural and Molecular Remodeling in Response to Osmotic Stress

**DOI:** 10.3390/cells12060858

**Published:** 2023-03-09

**Authors:** Océane Busont, Gaëlle Durambur, Sophie Bernard, Carole Plasson, Camille Joudiou, Laura Baude, Françoise Chefdor, Christiane Depierreux, François Héricourt, Mélanie Larcher, Sonia Malik, Isabelle Boulogne, Azeddine Driouich, Sabine Carpin, Frédéric Lamblin

**Affiliations:** 1 Laboratoire de Biologie des Ligneux et des Grandes Cultures, Université d’Orléans, INRAE, USC 1328, CEDEX 2, F-45067 Orléans, France; 2 GLYCOMEV UR 4358, SFR Normandie Végétal FED 4277, Innovation Chimie Carnot, University of Rouen Normandie, IRIB, F-76000 Rouen, France; 3 INSERM, CNRS, HeRacLeS US 51 UAR 2026, PRIMACEN, University of Rouen Normandie, F-76000 Rouen, France; 4 Department of Biology, University of Fribourg, CH-1700 Fribourg, Switzerland

**Keywords:** AC-DCs, mucilage, drought stress, root cap, xylogalacturonan, xylan, arabinogalactan protein, rhamnogalacturonan-I

## Abstract

The root extracellular trap (RET) consists of root-associated, cap-derived cells (root AC-DCs) and their mucilaginous secretions, and forms a structure around the root tip that protects against biotic and abiotic stresses. However, there is little information concerning the changes undergone by the RET during droughts, especially for tree species. Morphological and immunocytochemical approaches were used to study the RET of black poplar (*Populus nigra* L.) seedlings grown in vitro under optimal conditions (on agar-gelled medium) or when polyethylene glycol-mediated (PEG_6000_—infused agar-gelled medium) was used to mimic drought conditions through osmotic stress. Under optimal conditions, the root cap released three populations of individual AC-DC morphotypes, with a very low proportion of spherical morphotypes, and equivalent proportions of intermediate and elongated morphotypes. Immunolabeling experiments using anti-glycan antibodies specific to cell wall polysaccharide and arabinogalactan protein (AGP) epitopes revealed the presence of homogalacturonan (HG), galactan chains of rhamnogalacturonan-I (RG-I), and AGPs in root AC-DC cell walls. The data also showed the presence of xylogalacturonan (XGA), xylan, AGPs, and low levels of arabinans in the mucilage. The findings also showed that under osmotic stress conditions, both the number of AC-DCs (spherical and intermediate morphotypes) and the total quantity of mucilage per root tip increased, whereas the mucilage was devoid of the epitopes associated with the polysaccharides RG-I, XGA, xylan, and AGPs. Osmotic stress also led to reduced root growth and increased root expression of the *P5CS2* gene, which is involved in proline biosynthesis and cellular osmolarity maintenance (or preservation) in aerial parts. Together, our findings show that the RET is a dynamic structure that undergoes pronounced structural and molecular remodeling, which might contribute to the survival of the root tip under osmotic conditions.

## 1. Introduction

Drought is one of the most severe environmental stresses affecting plant growth and productivity [1]. The black poplar (*Populus nigra* L.) is an emblematic alluvial forest species that grows along large rivers with considerable seasonal flow variation. The tree is regularly exposed to periods of flooding, followed by periods of increasingly low water levels and thereby drought conditions. In the context of global warming, wild and cultivated poplars are being subjected to increasingly prolonged and intense periods of drought. The restoration and protection of riparian forests is one of the key priorities in biodiversity conservation and climate change adaptation. In this context, black poplar plays a major role in the development of riparian forests in European strategies [2]. The sexual regeneration of riparian forests using seedlings is essential for gene flow and in order to enhance disease resistance and adaptation to environmental changes through the natural genetic variation of populations [3]. However, the survival of poplar seedlings depends on the combination of many factors. After seed germination, the seedlings may face many life-threatening stresses such as drought in the summer [4,5]. During their first summer, poplar seedlings require a root growth rate of between 0.4 and 1.3 cm·day^−1^ in order to maintain contact with the alluvial water table [5].

Water and nutrients are provided to the aerial parts of plants by the roots. Indeed, plant roots and their exudates are in direct contact with the soil and play a key role in water and nutrient uptake [6]. Accordingly, this organ is considered the primary site for sensing drought and initiating signaling cascades to the rest of the plant, to ensure adequate responses [7]. However, little attention has previously been paid to the roots, even though they are on the frontline in terms of this stress. In such stressful environments, plants must preserve the root apical meristem (RAM) functional integrity, to maintain their growth. The RAM can be divided into three main zones. The meristematic zone at the root tip contains the stem cell niche and is also called the dividing zone. Cells which have left the meristematic zone following cell division belong to the elongation zone. The last zone contains cells that have acquired their destined cell fate, and corresponds to the differentiation zone [8]. During root growth in the soil, the RAM is protected by a root cap, which produces detached single cells (border cells), or organized layers of several cells attached to each other (border-like cells). These two types of cells can be regrouped and referred to as root-associated, cap-derived cells (root AC-DCs) [9,10,11,12,13]. Root AC-DCs are embedded in a mucilage layer that is mostly produced by these cells. This mucilage is an interface between the plant’s roots and the soil, and it has a protective role for roots and their meristem against biotic and abiotic stresses such as drought [14].

AC-DCs and their mucilaginous secretions constitute the root extracellular trap (RET) [12,13]. Different morphotypes of AC-DCs have been described in different species, depending on their size and shape [15,16]. The structure of the RET and the nature of the secreted molecules have now been studied in various herbaceous species such as Arabidopsis [11], pea [10,17], soybean [16,18], and maize [19], but in very few woody species [15,20]. Different analytical methods have shown that the mucilage secreted by AC-DCs contains proteins, including enzymes, antimicrobial proteins [18,21,22,23], malic acid [18], glycomolecules (pectins, arabinogalactan proteins, extensins, hemicelluloses, …) [16,17,24,25], and extracellular DNA [26,27,28].

A number of studies have highlighted the role of RET in root defense against microbial pathogens and in its interactions with beneficial microorganisms [16,17,18,29,30,31,32,33]. Less is known about its protective role against abiotic stresses, although it is known that these mucilaginous secretions participate in the detoxification of the root environment by trapping heavy metals (aluminum, arsenic, copper, cadmium, lead, mercury, iron, and zinc) [34,35,36].

During a drought, the root mucilage can absorb large volumes of water (due to its polysaccharide components), altering the physical properties of the rhizosphere and maintaining a wet and conductive environment in the rhizosphere when the soil dries. It is hypothesized that the mucilage acts as a hydraulic bridge between the roots and the soil, facilitating plant water uptake and maintaining transpiration in dry soils [6]. Most studies have focused on a range of grassland or crop species with different growth strategies [37], such as chickpea [38], barley [39], or maize [40].

Woody plant species with lower growth rates over a season are less sensitive to drought than those with a faster growth, such as grassland and crops [41]. Studies on AC-DCs and mucilage involvement in tree drought tolerance have hitherto been few in number. A comparison of three Sahelian woody species with contrasting sensitivities to drought stress revealed a link between polysaccharide composition and drought tolerance [15].

Seedling establishment is a critical phase in reforestation and forest preservation, due to a high mortality rate during juvenile stages. The role of AC-DCs and their mucilages in preserving root growth during droughts in crop species raises the question of a similar RET function in poplars during the early growth stages. Therefore, characterizing the RET of black poplar seedlings and the consequences of osmotic stress on both RET structural features and mucilage glycopolymer composition could shed light on their roles in the protection of meristem and subsequent seedling survival under drought conditions. The specific objectives of this study were (i) to characterize the root extracellular trap of black poplar seedlings for the first time, and (ii) to investigate the consequences of osmotic stress mimicking drought on the structural features of RET and the polysaccharide composition of the AC-DC cell walls and mucilage.

## 2. Materials and Methods

### 2.1. Plant Material

Given the difficulty of collecting AC-DCs and root mucilage in the soil, the majority of studies are carried out on young seedlings grown in vitro under axenic conditions [11,15,16,18]. Black poplar (*Populus nigra* L.) seeds were collected and stored at −20 °C, as described by Suszka et al. [42]. Seeds were harvested along the Loire, in the alluvial forest of the region of Orleans (France), in locations far enough from cultivated poplar plantations to avoid any hybridization. Seeds were surface-sterilized and sown on a Whatman filter paper placed in a square Petri dish (120 mm × 120 mm) filled with 35 mL ½ MS solid medium (supplemented with 20 g·L^−1^ sucrose; 200 mg·L^−1^ glutamine; 10 g·L^−1^ agar; pH 5.8). The Petri dishes were placed vertically in a growth chamber, at 24 °C under a 16 h photoperiod.

### 2.2. Application of an Osmotic Constraint on 3-Day-Old Seedlings

**Figure 1:**
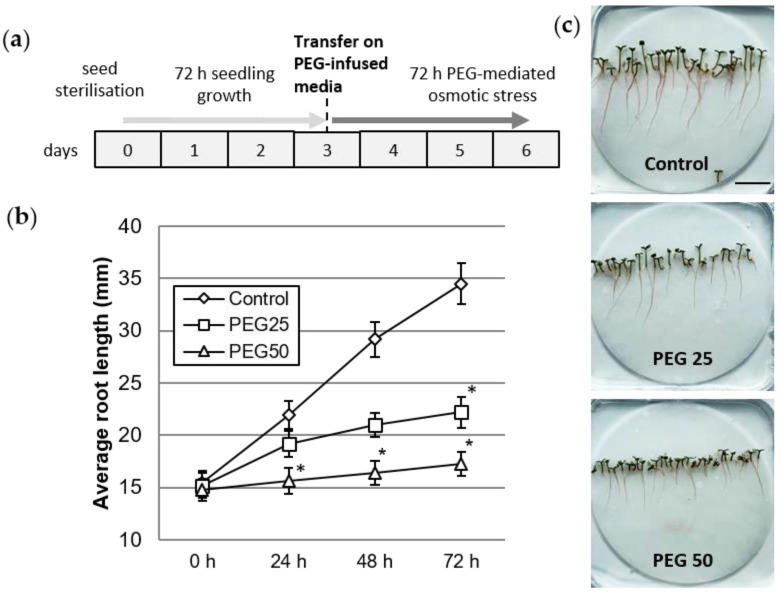
Plant growth under control and osmotic stress conditions. (**a**) Schedule of seedling culture and osmotic stress application. (**b**) Root growth kinetics of seedlings grown for 72 h under control and PEG-mediated osmotic stress conditions. Mean and standard error from six biological replicates (10 plants per replicate). * indicates significant difference with the control at level alpha = 0.05 according to Kruskal–Wallis test with Dunn’s procedure. (**c**) Six day-old seedlings after 72 h growth under control, moderate (PEG25), or strong (PEG50) osmotic stress conditions (bar scale: 2 cm).

Polyethylene glycol (PEG), a high molecular weight (6000 Da and more) non-permeable osmolyte, is commonly used to generate water stress conditions in vitro [43]. PEG infused into the agar-gelled medium effectively lowers the average water potential, and therefore disrupts water uptake by plant roots for a fixed period of time. Moreover, PEG-infused media limit the damage caused by hypoxia compared to hydroponics. We used PEG_6000_-infused agar-gelled media to grow young black poplar seedlings under drought stress conditions, according to the method described by Verslues [44]. Three types of PEG overlay solution were prepared, as follows: solution 1 (Control) consisted of ½ MS liquid medium; solution 2 (PEG25, moderate osmotic constraint) contained 100 mL ½ MS liquid medium + 25 g PEG_6000_; solution 3 (PEG50 strong osmotic constraint) contained 100 mL ½ MS liquid medium + 50 g PEG_6000_. After one night, the overlay solution was removed and the 3-day-old seedlings were transferred with their filter paper to these new media for a further 3-day cultivation period (Figure 1a). The osmolarity of the control, PEG25 and PEG50 infused agar media were respectively 123, 351, and 878 mosmoles·kg^−1^ of water (measured using a freeze point depression micro-osmometer) corresponding to a water potential of −0.31, −0.87 and −2.17 Mpa, respectively.

### 2.3. Plant Osmolarity Measurements

Water extracts were prepared by grinding aerial part tissues (cotyledons and hypocotyl segments) in ultrapure water (900 μL for 100 mg fresh tissue) in a mortar. The osmolarity of the supernatant collected after centrifugation (16,000× *g*, 5 min, 4 °C) was measured using a freeze point depression micro-osmometer.

### 2.4. Delta-1-Pyrroline-5-carboxylate Synthase 2 (P5CS2) Gene Expression Analysis

We monitored the expression of the P5CS2 gene encoding a Delta-1-pyrroline-5-carboxylate synthase 2 involved in the synthesis of proline [45]. Total RNA was extracted from 1 cm long root tips excised from 6-day-old seedlings grown under control and PEG-mediated osmotic stress conditions using a Macherey Nagel NucleoSpin RNA Plant kit. On-column RNAse free DNaseI treatment was carried out (15 min, room temperature) to remove genomic DNA. The reverse transcription was carried out using 1 μg of total RNA with a New England Biolabs ProtoScriptB II Reverse Transcriptase kit. The reaction mix was prepared in a 96-well reaction plate using Fast SYBR Green Master Mix (Applied Biosystems, Thermo Fisher Scientific, Strasbourg, France) in a final volume of 13 μL with 200 nM of each primer and 3 μL of cDNA template. The P5CS2 (Potri.010G198400.1, forward primer 5′ GTAGCTTTCTCACTTGAAG 3′, reverse primer 5′ CCTAACATACAGACTCGG 3′, amplicon size 97 bp), YLS8 (Potri.007G072700.1, forward primer: GGGTGCCCTGGTTAATTCAAAATCTC, reverse primer: CATGGAACTGGTTATCCACACCAACC, amplicon size 65 bp) PDF2 (Potri.010G127500.1, forward 5′-TAGCTAGTCAGACTCTTTGTAAGATTGG-3′, reverse 5′-CACTTCAGTACAACATGGGGTTCACC-3′, amplicon size 62 bp) genes were amplified using real-time PCR. Quantitative real-time PCR was performed using the CFX96 real-time system (Bio-Rad, Marnes-la-Coquette, France). The following parameters were used: 20 s at 95 °C then 40 cycles of 5 s at 95 °C, 20 s at 60 °C followed by the melt-curve analysis: 15 s at 95 °C, 6 min at 60 °C, and 15 s at 95 °C. Data were analyzed using CFX Maestro software 2.3 (Bio-Rad, Paris, France). The relative expression was normalized with the 2−∆∆Cq method to YLS8 and PDF2 as reference genes using GENorm analysis (provided in CFX Maestro software 2.3).

### 2.5. Root AC-DC Microscopic Observation and Mucilage Detection Assay

Root tips and root AC-DCs were examined for their ability to produce mucilage using India ink contrast staining on day 6, after 72 h under control or osmotic stress conditions. To visualize the RET, root tips (5 mm) were mounted directly between the slide and coverslip and a solution of India ink (1.5% in distilled water) was gently added by capillarity. After 5 min of rehydration of the mucilage, the roots were observed under a bright field inverted microscope Olympus IX73 (OLYMPUS S.A.S, Rungis, France). For further counting and characterization, the root AC-DCs were also isolated from the root tip by incubating the root in the well of a 10-well diagnostic slide for 5 min with 3 μL of 1.5% India ink solution, followed by 30 s gentle shaking, and then observed under a microscope.

### 2.6. Root AC-DCs and Root Tip Viability

Cell viability was assessed using a fluorescein diacetate (FDA) probe. Root AC-DCs, detached by gentle shaking of the root tip, were incubated for 5 min in a FDA solution (0.01 mg·mL^−1^ in phosphate buffer saline). For each morphotype, the percent viability was calculated using the following equation: % of AC-DCs viability = (number of fluorescent AC-DCs/total number of cells) × 100. For each root a minimum of 100 AC-DCs (all morphotypes combined) were examined. Root tips, after removal of the RET, were incubated for 5 min in FDA, as previously described by Ropitaux et al. [16]. Observations were made using an inverted epifluorescence microscope (Olympus IX73, bandpass excitation filter excitation 495/20 nm; bandpass emission filter 540/30 nm).

### 2.7. Image Analysis

Root length, root AC-DC length, width and surface, and mucilage surface were measured using Mesurim Pro software (J-F Madre, ENS, ACCES, Lyon, France). The mucilage was estimated by calculating the mucilage surface/cell surface ratio.

### 2.8. Statistical Analysis

Statistical analysis was performed with the statistical software XLSTAT (2018.1.1 software, Copyright Addinsoft 1995–2020), using a Kruskal-Wallis test followed by multiple pairwise comparisons using Dunn’s procedure at the significance level 0.05.

### 2.9. Resin Embedding Protocol on Root Tips with RET

Six day-old root tips with RET were embedded in 2.5% (*w*/*v*) low gelling temperature agarose in 0.1 M sodium cacodylate buffer (pH 7.2). Agarose blocks containing roots were processed at 4 °C with an electron microscopy tissue processor (EM-TP, Leica microsystems), as follows: Samples were fixed for 1 h 30 m in 1% paraformaldehyde and 1% glutaraldehyde mixture (*v*/*v*) in 0.1 M sodium cacodylate buffer (pH 7.2) and washed in distilled water. Samples were then dehydrated in ethanol, embedded in LRW resin, and polymerized for 48 h at 4 °C with UV light in LRW resin complemented with the UV catalyst benzoin methyl ether (0.5% *w*/*v*). Sections from resin blocks (2 µm; EM UC6 Leica microsystems, Nanterre, France) were collected on 10-well slides previously coated with poly-L-lysine (0.01% *v*/*v*).

### 2.10. Immunofluorescence Labeling on Resin Sections

Resin sections were blocked in PBS-T (phosphate buffered saline—Tween: NaCl 137 mM; KCl 2.7 mM; Na_2_HPO_4_ 10 mM; KH_2_PO_4_ 1.8 mM; Tween 20 0.1% (*w*/*v*)) supplemented with 3% of BSA (bovin serum albumin) and NGS 1/20 (normal goat serum, *v*/*v*) for 30 min. Sections were washed in PBS-T + 1% BSA and incubated overnight at 4 °C with the primary antibody (d: 1/2, see list in supplemental data Table S1 [46,47,48,49,50,51,52,53,54,55,56,57], Plant probes). After washing in PBS-T + 1% BSA, sections were incubated for 2 h at 25 °C with a secondary antibody coupled to Alexa 488 (d: 1/200, Invitrogen, Thermo Fisher Scientific, France). Finally, sections were washed in PBS-T + 1% BSA and ultra-pure water and observed using an epifluorescence microscope (Leica DMI6000B, with camera EL6000, Wetzlar, Germany), with a fluorescence filter cube L5 (BP 480/40) and an exposure time of 530 ms. Each immunolabeling was done with a negative control without the primary antibody and on three root tips per condition.

## 3. Results

### 3.1. Physiological Response of P. nigra Seedlings to Osmotic Stress

By day 3, the seedlings had developed a 15 mm primary root, hypocotyl, and green cotyledons. At that time, the seedlings were transferred onto control or PEG-infused media for a period of 72 h (Figure 1a). Root growth was significantly reduced upon application of osmotic stress, particularly under the PEG50 condition (Figure 1b). The average root growth rate was 6.3, 2.3, and 0.8 mm per day in the control, PEG25, and PEG50 treated seedlings, respectively. Figure 1c also shows the inhibited growth of the aerial parts (hypocotyl and cotyledons), compared to the control plants, proportionally to the osmotic stress intensity.

**Figure 2:**
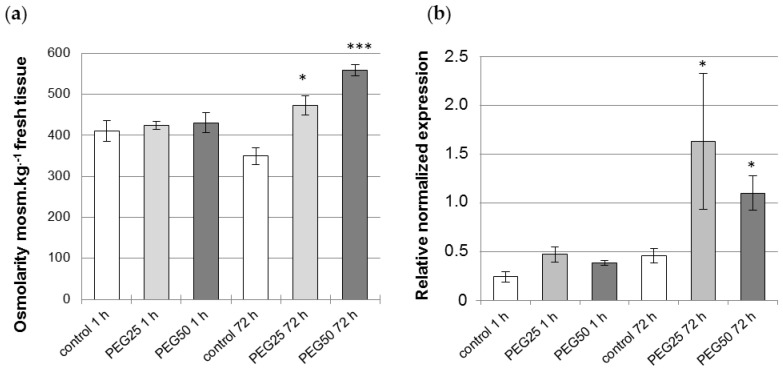
Physiological response of *P. nigra* seedlings to osmotic stress. (**a**) Osmolarity of aerial tissues (cotyledons, hypocotyl) of seedlings measured after 1 h and 72 h under control and PEG-mediated osmotic stress conditions. Mean and standard error from four biological replicates. (**b**) Transcript abundance of *P5CS2* gene in the root tips of seedlings grown for 1 h and 72 h under control and PEG-mediated osmotic stress conditions. Relative transcript levels of *P5CS2* were normalized to the abundance of the reference genes (*YSL8* and *PDF2*) transcripts. Mean ± standard error from three biological replicates. * and *** indicate a significant difference with the corresponding control at level alpha = 0.05 or 0.0001, respectively, according to a Kruskal–Wallis test with Dunn’s procedure.

The plant osmolarity did not change after 1 h in the control or when osmotic constraint was applied (Figure 2a), but increased significantly after 72 h, by 1.4- and 1.6-fold in comparison with the control in response to intermediate (PEG25) and strong (PEG50) osmotic stress, respectively. In the same way, *P5CS2* gene expression was also found to be significantly enhanced, by 3.5- and 2.4-fold, under intermediate and strong osmotic stress, respectively, as compared to the control (Figure 2b).

### 3.2. Light Microscopy Observation of P. nigra Root Extracellular Trap

**Figure 3:**
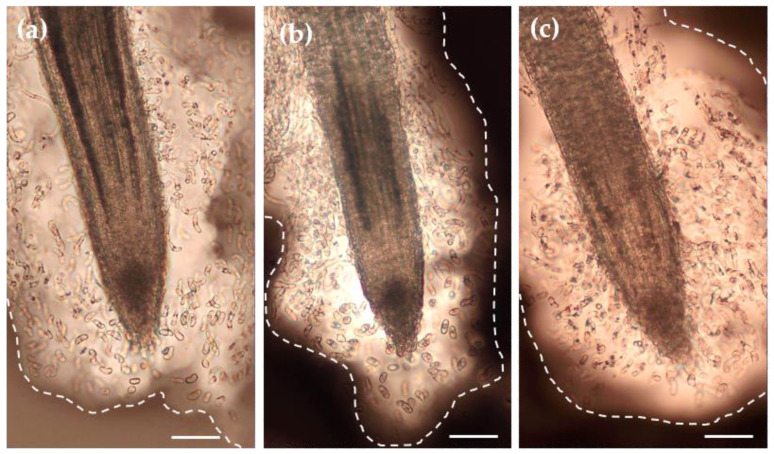
Microscopic observation of the RET of root tips of the control (**a**) and stressed (**b**,**c**, PEG25 and PEG50, respectively) seedlings using India ink contrast staining. Root tips were collected on day 6 after 72 h of osmotic stress. The images show a significant amount of mucilage (delimited by the dotted line) embedding AC-DCs. Bar scale: 100 μm.

As shown on Figure 3, the *P. nigra* RET contained only detached root AC-DCs. Irrespective of the condition, no layers of AC-DCs adhering to each other could be observed along the root tips. The control roots (Figure 3a) showed a RET where AC-DCs were embedded in a loose mass of mucilage when stained with India ink. The AC-DCs were observed at the apex and were present up to 1.5 mm along the root. Under osmotic stress conditions (Figure 3b,c), and particularly in the PEG50 condition, the RET formed a mass that was confined to the apical zone and rarely spread further than 0.8 mm from the root apex.

### 3.3. Characterization of Root AC-DC Morphotypes and Their Mucilage

**Figure 4:**
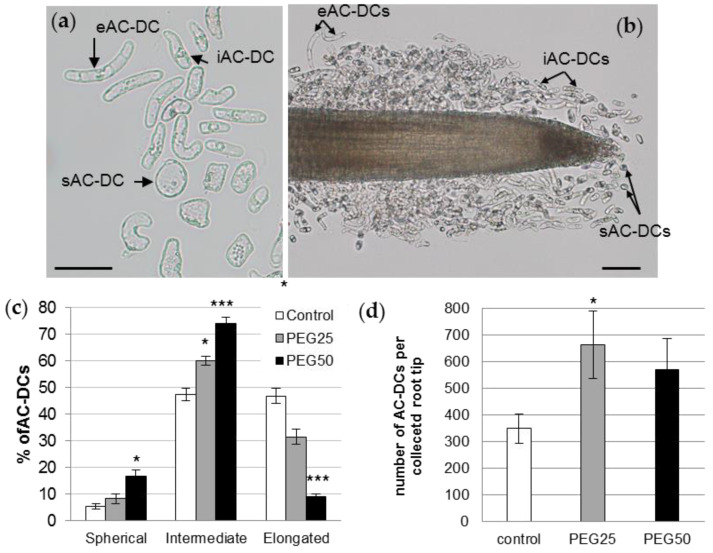
*P. nigra* root AC-DCs. (**a**) A microscopy image of AC-DCs collected on day 6 from a control root tip. Three different morphotypes were observed in the RET and characterized by their length to width ratio (R): sAC-DC, «spherical» (1 ≤ R < 2); iAC-DC, «intermediate» (2 ≤ R < 5.5); and eAC-DC, «elongated» (R ≥ 5.5); bar scale: 50 μm. (**b**) Location of the different AC-DC morphotypes along the root tip of a 6-day-old control plant; bar scale: 100 μm. (**c**) Relative proportions of different morphotypes of root AC-DCs collected from root tips after 72 h under control and osmotic stress conditions. (**d**) Total number of AC-DCs collected per root tip. Mean and standard error of 9 roots from 3 biological replicates. *: significant at level alpha = 0.05; ***: significant at level alpha = 0.0001 according to a Kruskal–Wallis test with Dunn’s procedure.

Bright field microscopic observations of AC-DCs isolated from the root apex showed a wide variety of shapes and sizes (Figure 4a). The ratios (R) of length to width were calculated on hundreds of AC-DCs collected from several root tips of control seedlings, and three cell morphotypes were designated as follows, according to the observations made previously by Ropitaux et al. (2020) on soybean: spherical (sAC-DC, 1 ≤ R < 2), intermediate (iAC-DC, 2 ≤ R < 5.5), and elongated (eAC-DC, R ≥ 5.5). Observation of the root tips of control seedlings revealed that the majority of the sAC-DCs were located at the root cap together with iAC-DCs, whereas the eAC-DCs were mainly distributed along the older part of the root (Figure 4b). The distribution of the different AC-DC morphotypes along the root tip was not modified in the stressed plants.

Regarding the proportion of each morphotype, the control root AC-DC populations consisted of 5.6% sAC-DCs, 47.5% iAC-DCs, and 46.9% eAC-DCs (Figure 4c). These proportions varied considerably in response to the strong osmotic stress (PEG50). The proportions of sAC-DCs and iAC-DCs increased significantly (16.9% and 73.9%, respectively), while eAC-DCs proportions decreased by 5-fold (9.2%) compared to the control. The application of moderate stress (PEG25) resulted in intermediate proportions of AC-DCs (8.3% sAC-DCs, 60.2% iAC-DCs, and 31.5% eAC-DCs). The average number of AC-DCs collected per root (all morphotypes together) was calculated for each of the experimental conditions. While an average of 350 AC-DCs was collected from the control roots, this number increased to 664 (significant at alpha = 0.05) and 570 under the PEG25 and PEG50 conditions, respectively (Figure 4d).

**Figure 5:**
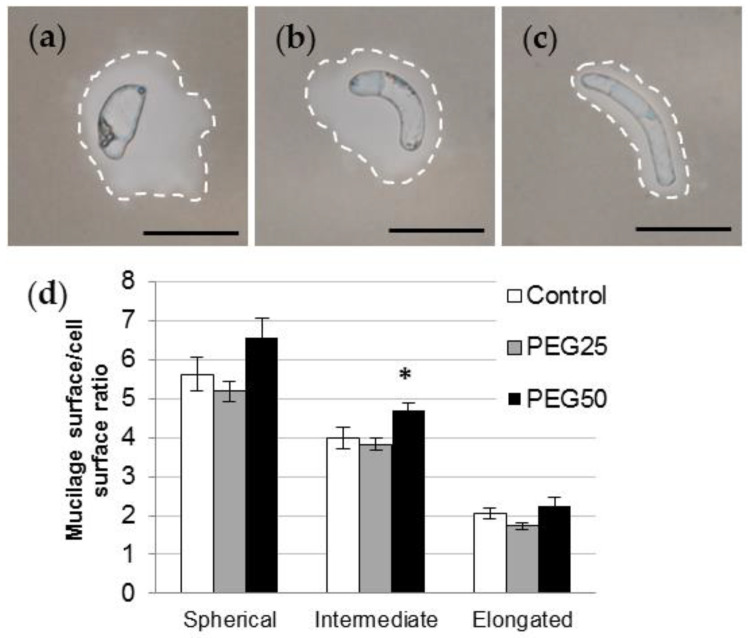
Mucilage secretion of *P. nigra* AC-DCs. India ink contrast staining showing spherical (**a**), intermediate (**b**), and elongated (**c**) AC-DCs collected on control root tips on day 6. Secreted mucilage is delimited by the dotted line. Bar scale: 50 μm. (**d**) Mucilage surface/cell surface ratio calculated on isolated spherical, intermediate, and elongated AC-DCs collected after 72 h (day 6) from control and stressed seedlings (mean and standard error from at least 100 cells collected from 10 root tips). * indicates significant difference with the control at level alpha = 0.05 according to a Kruskal–Wallis test with Dunn’s procedure.

India ink contrast staining revealed various amounts of mucilage secretions surrounding AC-DC morphotypes (Figure 5a–c). Regardless of the experimental conditions, the sAC-DCs produced significantly more mucilage than iAC-DCs and eAC-DCs, the latter exhibiting the thinnest layer. In control roots, the average ratio was 5.6, 4.0, and 2.1 for sAC-DCs, iAC-DCs, and eAC-DCs, respectively (Figure 5d). Under moderate stress, the quantity of mucilage produced was the same as the control for all of three morphotypes studied, whereas a slight increase in mucilage content was observed under strong stress conditions (significant at *p* = 0.05 for the iBCs, Figure 5d).

### 3.4. Viability of Root AC-DCs and Meristematic Zone under Osmotic Stress

**Figure 6:**
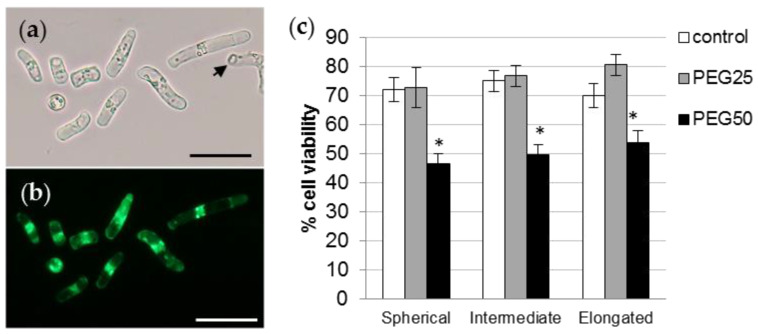
Viability of *P. nigra* root AC-DCs. Isolated AC-DCs from control seedlings were observed under bright field (**a**) and fluorescence (**b**) after staining with the FDA probe. The arrow shows a non-fluorescent dead cell. Bar scale: 50 μm. (**c**) Percentage of viability of the different AC-DCs morphotypes after 72 h under control and stress conditions (mean and standard error of 12 roots from 4 biological replicates, with a minimum of 100 AC-DCs per root). * indicates significant difference with the corresponding control at level alpha = 0.05 according to a Kruskal–Wallis test with Dunn’s procedure.

AC-DC viability was analyzed using FDA probe fluorescence (Figure 6a,b). Under control conditions, the percentage of cell viability (Figure 6c) was at least 70% (72.3, 75.2, and 70% for sAC-DCs, iAC-DCs, and eAC-DCs, respectively). The viability of AC-DCs was not affected by the application of moderate stress (PEG25), but was significantly reduced to approximately 50% for all morphotypes after 72 h under conditions of strong osmotic stress (PEG50) (46.5, 49.7, and 54% for sAC-DCs, iAC-DCs, and eAC-DCs, respectively).

**Figure 7:**
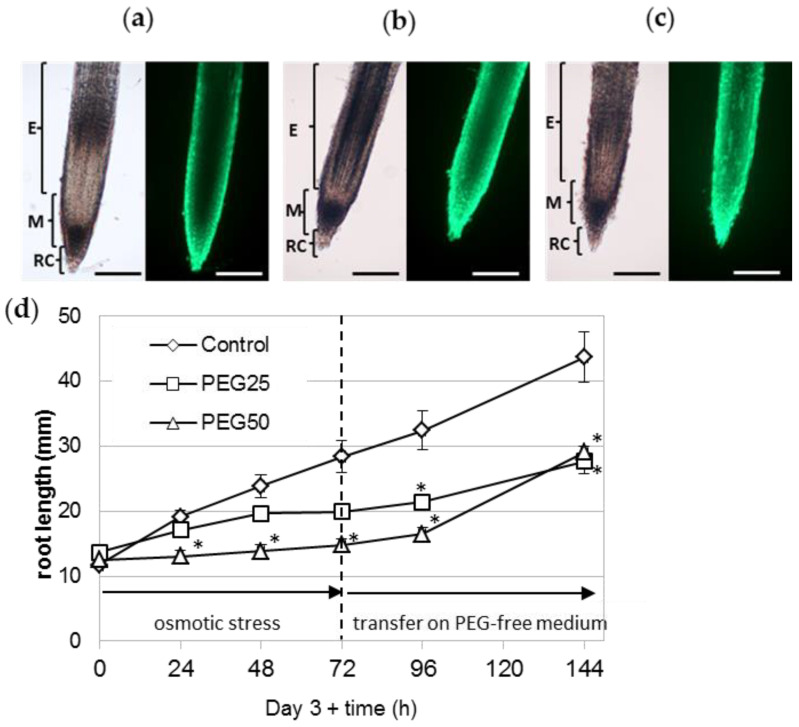
Survival of the root apical meristem under osmotic stress. The viability of the root tip (meristematic and elongating zones) of seedlings grown for 72 h under control conditions (**a**), moderate (**b**, PEG25), and strong (**c**, PEG50) osmotic stress was assessed using the FDA probe. The RET was removed beforehand, to facilitate observation of the root tip. Bar scale: 200 μm. E, elongation zone; M, meristematic zone; RC, root cap. (**d**) Recovery of stressed seedlings’ root growth after transfer back to ½ MS control medium. After 72 h of osmotic stress, seedlings were transferred onto a fresh ½ MS control medium without PEG (mean and standard error of 10 roots from one biological replicate). * indicates significant difference with the corresponding control at level alpha = 0.05, according to a Kruskal–Wallis test with Dunn’s procedure.

The survival of the root tip (meristematic and elongating zones) was also assessed using the FDA probe. Detailed observations of several roots under the microscope showed that a large number of cells from the root cap, meristematic zone, and elongation zone were viable, regardless of the experimental condition (Figure 7a–c). The survival of the root tip was also evidenced by transferring 6 day-old stressed seedlings to ½ MS control medium for a further 72 h period (Figure 7d). Stressed seedlings regained a growth rate nearly comparable to that of the control seedlings (5.1, 2.5, and 4.8 mm per day for control, PEG25 and PEG50 stressed seedlings, respectively).

### 3.5. Immunocytochemical Characterization of Glycopolymers in Control RET and Root Tip

**Figure 8:**
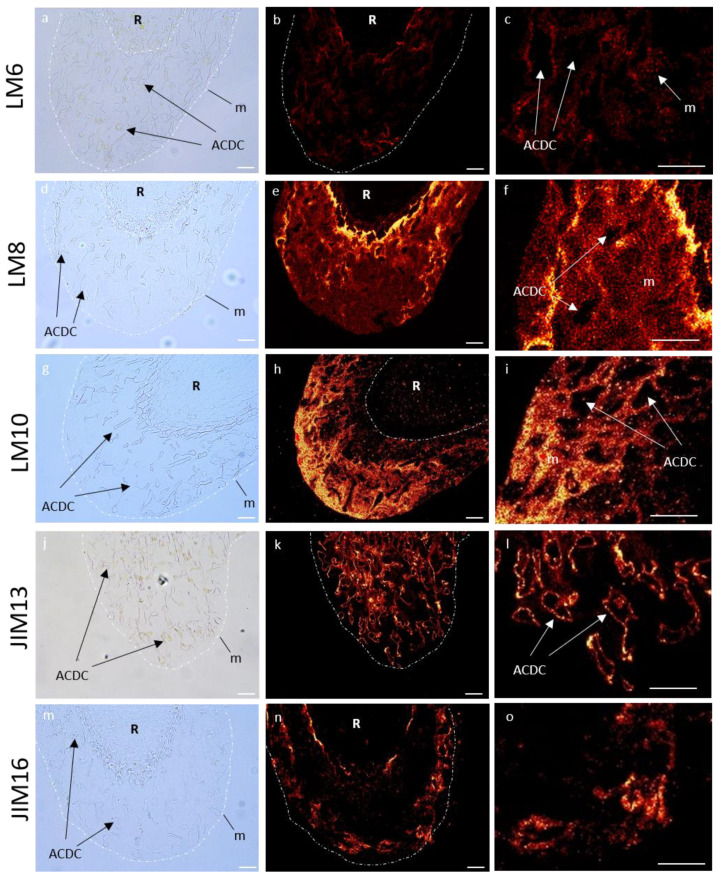
Immunocytochemical characterization of glycopolymers of control *P. nigra* root extracellular trap. LM6 branched arabinan side chains from rhamnogalacturonan 1 (**a**–**c**), LM8 xylogalacturonan (**d**–**f**), LM10 xylan (**g**–**i**), JIM13 arabinogalactan protein (**j**–**l**), and JIM16 arabinogalactan protein (**m**–**o**). Left column: bright field images defining mucilage and AC-DC, central column: corresponding images with fluorescence, right column: zoom of fluorescence images. AC-DC: root associated cap derived cell, m: mucilage, R: root. Bars: 25 µm.

The occurrence of cell wall polysaccharides (pectins and hemicelluloses) and hydroxyproline rich glycoproteins (arabinogalactan proteins and extensin) was investigated on resin-embedded longitudinal sections of root tips, using specific monoclonal antibodies (mAbs, see list Table S1 in Supplemental Data). For each antibody, we observed the fluorescence signal of the RET (mucilage and root associated cap-derived cells (AC-DCs)) and the root (meristematic and elongation zones). The data are summarized in Table 1, and images are shown in Figure 8 and Figures S1–S4.

#### 3.5.1. Immunolocalization of Pectin Epitopes

In the RET, weakly esterified homogalacturonans (HG) (detected with LM19) showed no fluorescence signal (Figure S1). Highly esterified HGs (LM20) were detected in AC-DC cell walls, but not in the mucilage (Figure S1). Arabinan chains from RG-I (LM6) were observed as fluorescent spots only in the mucilage (Figure 8), and galactan chains (LM5) were only found in AC-DC cell walls (Figure S1). Xylogalacturonan (XGA) epitopes (LM8) were strongly detected in the mucilage in the area nearest the root but less frequently detected in the rest of the mucilage, forming fluorescent spots (Figure 8), contrary to the AC-DC cell walls, where no fluorescence was detected.

Cell walls from meristematic and elongation zones showed a strong labeling with LM19 (Figure S1) and a weak labeling with LM20 (Figure S1) and LM6 (Figure S1). LM5 was only detected in the elongation zone (Figure S1) and LM8 was not detected at all (Figure S1).

#### 3.5.2. Immunolocalization of Hemicellulose Epitopes

Neither XyG epitopes (LM25) nor those associated with heteromannan (LM21) were detected in the RET (Figure S2). Unsubstituted and relatively low substituted xylan recognized by LM10 were only labeled in the mucilage, with a weak intensity in the area nearest the root and a stronger intensity in the outer area of the mucilage forming fluorescent spots (Figure 8).

Cell walls from meristematic and elongation zones presented a fluorescent signal for LM25, but not for LM21 and LM10 (Figure S2).

#### 3.5.3. Immunolocalization of Hydroxyprolin Rich Glycoprotein (HRGP) Epitopes

We used four antibodies (JIM8, JIM13, JIM14, and JIM16) to identify different arabinogalactan proteins (AGPs) epitopes. With JIM13, we observed a fluorescence signal in the RET restricted to the root AC-DC cell walls, and not in the mucilage (Figure 8). The same observation was made with JIM14, with a weaker fluorescence signal (Figure S3). On the contrary, we observed a strong fluorescence signal in the mucilage with JIM16, which seemed to be restricted to the peripheral area (Figure 8). No fluorescence was detected with JIM8 (Figure S3). Extensin epitopes recognized by JIM12 and LM1 were not detected (Figure S4).

Cell walls from meristematic and elongation zones only presented labeling for JIM8 and JIM13 (Figure S3). JIM14, JIM16, or extensin epitopes recognized by JIM12 and LM1 showed no fluorescence signal (Figures S3 and S4).

In summary, in control conditions, mucilage of *P. nigra* RET was characterized by labeling of branched arabinan side chains by RG-I (LM6), XGA (LM8), xylan (LM10), and AGPs (JIM16) (Figure 8), whereas highly esterified HG (LM20), galactan chains (LM5), and AGPs (JIM13 and weakly JIM14) were restricted to the AC-DC cell walls.

### 3.6. Immunocytochemical Characterization of Glycopolymers in RET and Root Tips under Osmotic Stress

After immunocytochemical characterization of glycopolymers on the root tip under control conditions, we investigated the potential remodeling of their labeling in root tips grown under strong osmotic stress (PEG50) (Table 2).

**Figure 9:**
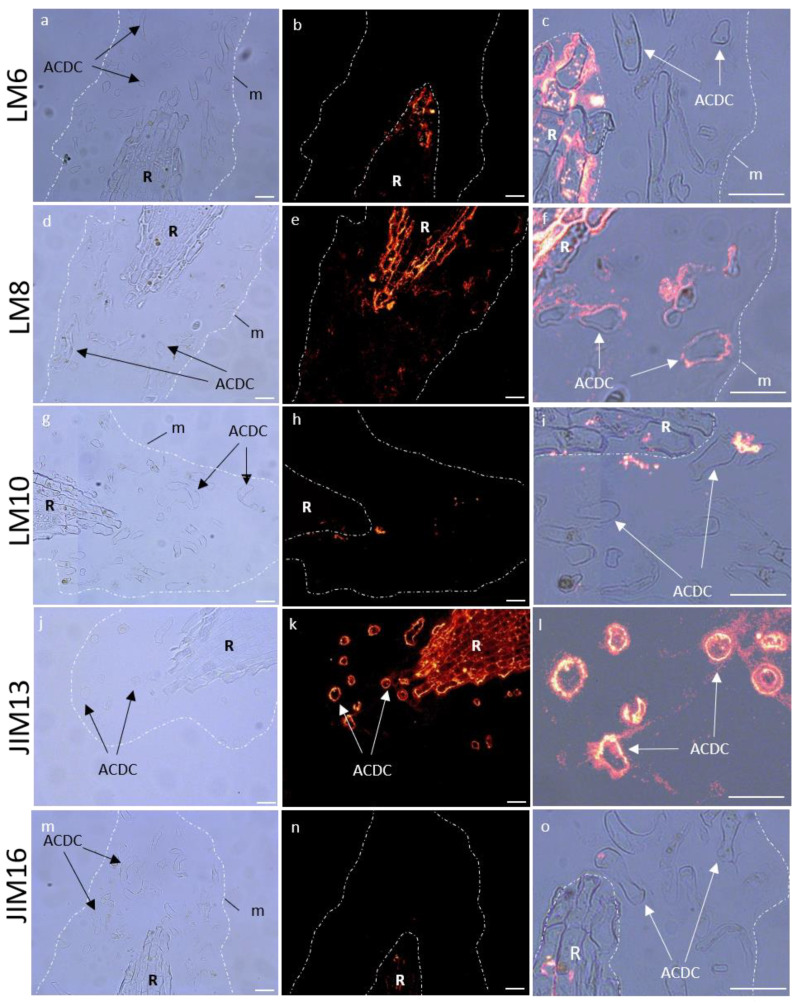
Immunocytochemical characterization of glycopolymers of *P. nigra* root extracellular trap under strong osmotic stress (PEG50). LM6 branched arabinan side chain from rhamnogalacturonan 1 (**a**–**c**), LM8 xylogalacturonan (**d**–**f**), LM10 xylan (**g**–**i**), JIM13 arabinogalactan protein (**j**–**l**), and JIM16 arabinogalactan protein (**m**–**o**). Left column: bright field images defining mucilage and AC-DC, central column: corresponding images with fluorescence, right column: zoom overlay of bright field/fluorescence images. The mucilage labeling of LM6, LM10, and JIM16 was not detected under osmotic stress, and lightly detected with LM8. AC-DC cell wall showed a strong labeling with JIM13. AC-DC: root associated cap derived cell, m: mucilage, R: root. Bars: 25 µm.

In RET, pectin labeling seemed to be highly affected, as we observed a complete loss of the fluorescence signal in the mucilage for LM6 and LM8 (Figure 9), and a significant decrease of LM20 and LM5 labeling in the cell wall of AC-DCs (Figure S5). On the contrary, the cell walls of meristematic and elongation zones showed an increase in LM20 and LM6 fluorescence signals, but a decrease in LM5, specifically in the elongation zone (Figure S5). For hemicelluloses, the mucilage of the RET showed a disappearance of LM10 signal (Figure 9), whereas an increase in LM25 epitopes (Figure S6) was observed in AC-DC cell walls.

The cell walls of meristematic and elongation zones showed a decrease in the fluorescence signal with LM25 (Figure S6).

For AGPs, we observed a loss in fluorescence signaling in the mucilage for JIM16 (Figure 9), whereas AC-DC cell walls presented an increase in JIM13 (Figure 9) and a decrease in JIM14 (Figure S7). Cell walls of meristematic and elongation zones showed an increase in JIM8 and JIM13 (Figure S7). No modification was detected for extensin in the RET or in the cell wall of the meristematic and elongation zones (Figure S8).

## 4. Discussion

### 4.1. The First Characterization of P. nigra RET and Root Tip Glycopolymers

The *P. nigra* root cap only released individual AC-DCs (so-called border cells) and no layers of AC-DCs adhering to each other (the so-called border-like cells). As suggested by Carreras et al. [15], the production of individual AC-DCs could be related to the organization of an open RAM. Indeed, similarly to soybean or pea (Fabaceae family), the RAM of the Salicaceae species is of the open type [58]. Under our experimental conditions, a mean of 350 AC-DCs were able to be collected on the control root tips, which is relatively low compared to other species such as maize [19] (several thousand AC-DCs per root tip) and Sahelian tree species [15], but much more than for Solanaceae species [59]. However, the dynamics of AC-DC production by the root cap of *P. nigra* seedlings was sustained. Indeed, after having been mechanically removed from the root tip, the RET was completely reconstituted in less than 24 h. 

Poplar RET collected from control roots contained a very low proportion of spherical AC-DCs (5.6%) and equivalent proportions of intermediate and elongated AC-DCs (47.5% and 46.9%, respectively). These results are in agreement with those observed for soybean [16], *B. aegyptiaca*, *A. raddiana,* and *T. indica* [15]. For *B. aegyptiaca*, *A. raddiana,* and *T. indica,* the intermediate AC-DCs were more prominent (more than 50%) compared to the spherical ones (less than 20%). The surface of mucilage detected around each AC-DC was significantly higher for spherical AC-DCs recently detached from the root cap compared to intermediate and elongated ones, as already observed in soybean [16]. This may reflect a higher quantity of mucilage being secreted by this morphotype in black Poplar.

Regarding studies on the primary cell wall and extracellular glycopolymer composition, the literature is lacking for the root tips (including the RET) of woody species at a very early stage of growth. For the first time, we characterized the glycopolymers present in the root tip of black poplar seedlings.

Our results showed that in 6-day-old *P. nigra* root tips, we can detect HG, RG-I, XyG, and AGPs in the cell wall of meristematic and elongation zones. Marzec-Shmidt et al. [60] studied cell wall formation, including the primary cell wall, in 3-month-old *Populus trichocarpa* roots, and in doing so detected HG, RG-I, XyG, AGPs, and extensin. Our data are consistent with these results, except in the case of extensin detection.

Root AC-DCs and mucilage showed different profiles regarding the glycopolymer epitopes detected. AC-DC cell walls presented HG, galactan chains of RG-I, and AGP epitopes, whereas the mucilage showed the presence of XGA, xylan, and AGP epitopes (and a low level of arabinan epitopes). Studies on RET mucilage using immunocytochemical characterization of glycopolymers have been performed on different plant models such as arabidopsis [11,24,61], flax [24], pea [17,62,63], soybean [16], potato [30], rapeseed [17], and sahelian woody plants [15]. Depending on the plant model and the antibodies used, the studies clearly showed the occurrence of different classes of pectins (HG, RG-I, XGA), hemicelluloses (XyG and mannan), and glycoproteins (AGPs and extensin). These studies did not test the presence of xylan, but a small amount of xylan was detected in extracellular glycopolymers from suspension-cultured cells of *Populus alba* [64]

Interestingly, we noticed a gradient in the signal intensity for XGA, xylan, and AGPs in the mucilage (see Figure 8). The distribution of these glycopolymers was not homogenous within the mucilage, and the area near the root tip and the outer area seemed to have specific glycopolymer localization. Most immunocytochemical studies of RET mucilage glycopolymers are based on surface immunolabeling, and therefore the inner mucilage epitopes cannot be detected. However, mucilage organization with different layers of specific glycopolymers was observed in seed coat secretions in different plant species, including arabidopsis [65,66], flax [67], and *Plantago ovata* [68].

Our results showed that the outer area of the mucilage mostly contained AGP, xylan, and RG-I, whereas the inner area had XGA and RG-I. Some studies support that these polymers can be linked together. Tan al. [69] identified an AGP from arabidopsis suspension cultured cells, covalently attached to RG-I and arabinoxylan. Moreover, they demonstrated a covalent link between RG-I and arabinoxylan. It was also proposed that pectins are linked to xylan through RG-I in tomato primary cell walls [70] and in arabidopsis seed coat mucilage [66]. Additionally, Coenen et al. [71] demonstrated that, in apple trees, XGA was covalently linked to RG-I.

AGPs were detected in both mucilage and root AC-DC cell walls, as well as in the cell walls of meristematic and elongation zone cells, but the detected epitopes were different (see Table S1). Our results show that the JIM16 epitope was only detected in the mucilage, the JIM14 epitope was found on AC-DC cell walls, and the JIM13 epitope was present on both root tips and AC-DC cell walls, but not in the mucilage. Therefore, a fine-tuned spatial distribution of different types of AGPs seems to occur within the RET of black poplar, but further investigations are needed in order to identify how this is controlled.

### 4.2. Physiological, Structural, and Molecular Remodeling in Response to Osmotic Stress

As observed in Arabidopsis [72], poplar seedlings were able to maintain a very low root growth under high osmotic stress, possibly by lowering their water potential and cell turgor. As proline is an important osmoprotectant, which accumulates in plants subjected to hyperosmotic stresses, including drought [73] or high salinity, the *P5CS2* gene encoding a Pyrroline-5-Carboxylate Synthetase involved in the synthesis of proline can be considered as a marker of osmotic stress response [45]. The root expression of the *P5CS2* gene and the global osmolarity of the tissues of the aerial parts both increased significantly after 72 h of strong osmotic stress, suggesting an osmotic adjustment and probably proline synthesis in the roots of poplar seedlings, in order to maintain water uptake. Resumption of the growth of stressed seedlings after transfer to the control medium demonstrated the maintenance of root meristem integrity during osmotic stress. A decrease in root growth rate was also observed in hybrid poplar plants (*P. deltoides* x *P. nigra*, cv ‘Soligo’) grown in a hydroponic culture and subjected to short-term PEG_4000_-mediated osmotic stress [74]. The authors reported that osmotic stress did not affect the RAM length as a result of homeostasis maintenance in short- to middle-term responses. Root growth inhibition was a consequence of decreased cell expansion throughout the growth zone.

Regarding the glycopolymer composition in the high osmotic stress condition (PEG50), we observed fluorescence signal modifications (increase or decrease) in the meristematic and elongation zones, as well as in AC-DC cell walls and mucilage (see Table 2).

Under drought stress, cell wall remodeling is essential, as cells need to increase their cell wall elasticity/plasticity to keep the cell wall turgid and to restrict growth by retarding cell extension [75]. In addition, Moore et al. [76] stated that drought tolerance appears to result in both the tightening and loosening of cell walls. Tissues that must be maintained in a growth “ready” state are loosened, whereas non-essential tissues are tightened.

In our study, the meristematic and elongation zones of the root tip presented an increase in highly esterified HG and highly branched RG-I arabinan side chains, and the disappearance of RG-I galactan side chains, which were only present on the elongation zone. To adapt to stress conditions, plants can change the degree of pectin methylation in the cell wall, due to a balance between pectin methylesterases (PME) and pectin methylesterase inhibitors (PMEI) [77], as has been observed in the roots of *Pisum sativum* [62] and *Beta vulgaris* [78] under drought stress. When the amount of esterification increases, the interactions between pectin and calcium to form gels decrease. Increasing the degree of RG-I branching to maintain cell wall plasticity with drought stress seems to be a common process [75]. For example, in wheat, Leucci et al. [79] demonstrated that the amount of RG-I side chains in the cell wall significantly increased in response to water stress in the roots of a drought-tolerant cultivar, but not in a drought sensitive one. Klaassen and Trindade [80] observed that degradation of RG-I galactan side-chains altered the water binding capacity of the cell wall, presumably by affecting the polysaccharide architecture (spacing) and interactions in the matrix.

The meristematic and elongation zones of the root tip also showed a decrease in XyG epitopes. XyG is a hemicellulose cell polysaccharide that cross-links cellulose microfibrils, to form a network essential for the structural integrity of the cell wall. XyG can also bind to RG-I [81]. Therefore, to keep cell wall plasticity under stress, XyG remodeling may occur in the cell wall in response to osmotic stress via the action of XyG-modifying enzymes. XyG endotransglycosylase/hydrolases (XTH) are enzymes that can disrupt and reconnect the XyG chains and are upregulated in Salicornia [82] and tomato plants [83] during drought stress.

Lastly, the meristematic and elongation zones of the root tip presented an increase in AGP epitopes detected by JIM8 and JIM13. Mareri et al. [84] hypothesized that AGPs form a “buffer zone” that prevents the direct interaction between the plasma membrane and cell wall matrix, and thus stabilizes the membrane structure during drought stress.

Regarding the RET, the mean number of *P. nigra* AC-DCs collected per root tip was increased by 1.9- and 1.6-fold under moderate and strong osmotic stress conditions, respectively. Similarly, the addition of PEG_6000_ (15% to moisten filter paper) to maize seedlings for 72 h led to reduced primary root length, whereas the total number of AC-DCs collected per root tip was significantly increased, by 1.6-fold [19]. In rice, the number of AC-DCs per root tip increased by up to 43% in response to NaCl (60 mM) treatment in the salt-tolerant Pokali variety but not in the salt-sensitive IR29 variety. The thickness of the mucilage layer surrounding the AC-DCs remained unchanged in Pokali but was decreased by 35% in IR29 in response to salt stress [85]. The authors concluded that, together with root growth parameters, AC-DCs represented a promising cellular marker for the screening of salt stress tolerant rice varieties. These data and our study suggest that AC-DCs and their mucilaginous secretions have an important role in reducing adversity damages to roots under osmotic stress conditions.

The relative proportions of the different morphotypes of AC-DCs changed significantly in response to strong osmotic stress, with a dramatic increase in spherical AC-DCs and intermediate AC-DCs, and a decrease in elongated AC-DCs. These elongated cells may be produced by lateral root cap-epidermal initials of the open type RAM, localized in the elongation zone [46]. However, as described by Bizet et al. [74], the length of the growth zone of poplar roots was significantly reduced in response to osmotic stress. It can be hypothesized that the application of osmotic stress for 72 h could decrease the turgor pressure known to play a role in cell elongation, and lead to a decrease in the length of this zone, and consequently a decrease in the number of elongated AC-DCs cells produced.

Secretion of mucilage by *P. nigra* AC-DCs remained unchanged under moderate osmotic stress but was slightly increased under high osmotic stress. However, by integrating all the data (average number of AC-DCs collected per root, relative proportions of the three morphotypes, and the mucilage surface/cell surface ratio), we estimated that the global quantity of mucilage surrounding the root apex and meristem and produced by all AC-DCs was respectively 1.6 times and 2.2 times higher under moderate and strong osmotic stress conditions in comparison with the control condition.

Root mucilage secreted into the soil could play a role in maintaining water uptake under water stress conditions. The mucilage is highly hydrophilic due to its polysaccharide composition (more than 78% [14]) and can absorb up to 300-times its dry weight in water [6]. Ahmed et al. [86] demonstrated that water retention was increased in a sandy soil supplemented with 1.25% mucilage in comparison to the control soil. Under drought, water depletion around the roots reaches a critical point, at which the soil conductivity becomes too low. The presence of mucilage helps to maintain the humid conditions of the rhizosphere and allows a longer period of water uptake [6]. Mucilages also play a role in the formation of rhizosheaths by acting as a glue between soil particles. Rabbi et al. [38] observed that drought tolerant chickpea varieties produced more root mucilage and formed greater and more porous rhizosheaths than sensitive ones. Nazari et al. [40] studied the composition and quantity of mucilage exuded by maize from contrasting climatic regions and found that Indian and Kenyan genotypes from semi-arid regions secreted 135 and 125% more mucilage, respectively, compared to genotypes from central Europe. Similarly, India ink staining showed that root tips of drought-tolerant barley genotypes produced 1.2- to 1.5-times more mucilage than sensitive genotypes [39]. In addition, the secretion of mucilage provides both a biofilm-like environment and an accessible carbon source, which could help maintain beneficial microbial communities in the rhizosphere during a drought [87,88].

Young *P. nigra* seedlings growing on sandy soils in alluvial areas are regularly subjected to periods of drought due to water table recession during the summer and have a very low survival rate during their first year (pioneer phase), despite a significant root growth rate (0.4–1.3 cm·day^−1^) [5]. Indeed, seedlings do not tolerate a water table recession exceeding 0.5 cm·day^−1^. In such sandy soils subjected to drought conditions, AC-DC-mediated secretion of mucilage creates a wet microenvironment in the rhizosphere and helps maintain the integrity and survival of the meristematic zone.

Contrary to the meristematic and elongation zones, which seem to modify their polysaccharide composition in order to loosen their cell walls, root AC-DCs in the RET seem to tighten them to a certain extent. Indeed, we observed a decrease in highly esterified HG and the AGP epitopes recognized by JIM14 and an increase in XyG. Concerning the RET mucilage, we observed a complete loss of signaling for the AGP, xylan, and RG-I epitopes present in the outer area, as well as the XGA and RG-I epitopes in the inner area. As these polysaccharides were shown to bind to each other [66,69,70,71], it is possible that the mucilage becomes looser under drought stress, which then reduces the detection of these specific polysaccharides. It is also possible that a strong remodeling between these polysaccharides occurs during osmotic stress, thereby masking these epitopes. Lastly, another hypothesis would be that black Poplar roots and AC-DCs decrease their secretion of these polymers during drought stress. Further monosaccharide analysis in the RET and roots of *P. nigra* using gas chromatography may help us to understand this issue.

## 5. Conclusions

This study provides the first description of the root extracellular trap of a model tree species from a temperate alluvial forest: the black poplar *Populus nigra*. Previously, the only data available for woody plants were for *Balanites aegyptica*, *Acacia raddiana,* and *Tamarindus indica,* which are species from the Sahelian regions [15], as well as *Acacia mangium* from the tropical regions [20]. Application of osmotic stress mimicking drought to *Populus nigra* seedlings had a significant impact on the primary root growth and the affected RET organization and structure, as well as provoking the remodeling of cell wall polysaccharides in the root tip, mucilage, and AC-DCs. This suggests that the mucilaginous “tissue” plays an important role in osmotic stress tolerance. Although the development of a biological model using single-cell omics technology would be challenging, [89], it could shed light on the metabolic pathways differentially expressed in the morphotypes of AC-DCs under normal and osmotic stress conditions.

## Figures and Tables

**Table 1:** Immunocytochemical characterization of glycopolymers of *P. nigra* RET and root tips. AC-DC: root associated cap derived cell, HG: homogalacturonan, RET: root extracellular trap, RG1: rhamnogalacturonan I, XGA: xylogalacturonan, XyG: xyloglucan, Het: heteromannan. (++) strong labeling; (+) labeling; (±) weak labeling, (−) no labeling.

			RET		Root	
			Mucilage	AC-DC	Meristematic Zone	Elongation Zone
Pectins	HG	LM19	−	−	++	++
		LM20	−	+	±	±
	RG-I	LM6	+	−	±	±
		LM5	−	+	−	++
	XGA	LM8	++	−	−	−
Hemicelluloses	XyG	LM25	−	−	+	+
	Het	LM21	−	−	−	−
	Xylan	LM10	++	−	−	−
Hydroxyprolin Rich Glycoproteins	Arabino- galactan Protein	JIM8	−	−	+	+
		JIM13	−	+	+	+
		JIM14	−	±	−	−
		JIM16	++	−	−	−
	Extensin	JIM12	−	−	−	−
		LM1	−	−	−	−

**Table 2:** Modification of glycopolymer immunolabeling in *P. nigra* RET and root tips in response to osmotic stress. AC-DC: root associated cap derived cell, HG: homogalacturonan, RET: root extracellular trap, RG-I: rhamnogalacturonan I, XGA: xylogalacturonan, XyG: xyloglucan, Het: heteromannan. =: Same labeling under osmotic stress, ↗: increased labeling under osmotic stress, ↘ decreased labeling under osmotic stress, ↘↘: loss of labeling under osmotic stress, white square: no labeling either under control conditions or under osmotic stress conditions.

			RET		Root	
			Mucilage	AC-DC	Meristematic Zone	Elongation Zone
Pectins	HG	LM19			=	=
		LM20		↘	↗	↗
	RG-I	LM6	↘↘		↗	↗
		LM5		↘		↘
	XGA	LM8	↘↘			
Hemicelluloses	XyG	LM25		↗	↘	↘
	Het	LM21				
	Xylan	LM10	↘↘			
Hydroxyprolin Rich Glycoproteins	Arabino- galactan Protein	JIM8			↗	↗
		JIM13		↗	↗	↗
		JIM14		↘		
		JIM16	↘↘			
	Extensin	JIM12				
		LM1				

## Data Availability

Not applicable.

## Supplementary Materials

The following supporting information can be downloaded at: https://www.mdpi.com/article/10.3390/cells12060858/s1, Table S1: Monoclonal antibodies used in immunocytochemical characterization of glycopolymers in RET (mucilage and AC-DCs) and root (meristematic and elongation zones) under control and strong osmotic stress. Figure S1: Immunocytochemical characterization of pectins in a control *P. nigra* root tip. Figure S2: Immunocytochemical characterization of hemicelluloses in a control *P. nigra* root tip. Figure S3: Immunocytochemical characterization of arabinogalactan proteins in a control *P. nigra* root tip. Figure S4: Immunocytochemical characterization of extensins in a control *P. nigra* root tip. Figure S5: Immunocytochemical characterization of pectins in a *P. nigra* root tip under osmotic stress. Figure S6: Immunocytochemical characterization of hemicelluloses in a *P. nigra* root tip under osmotic stress. Figure S7: Immunocytochemical characterization of arabinogalactan proteins in a *P. nigra* root tip under osmotic stress. Figure S8: Immunocytochemical characterization of extensins in a *P. nigra* root tip under osmotic stress.
