# Supplementary material for: Black Poplar (Populus nigra L.) Root Extracellular Trap, Structural and Molecular Remodeling in Response to Osmotic Stress
Source: Cells. 2023 Mar 9;12(6):858. doi: 10.3390/cells12060858 (PMC10047092; doi:10.3390/cells12060858)
Supplement: Supplementary file 1 [file cells-12-00858-s001.zip › cells-2257009-supplementary.pdf]

cells

Busont et al. Supplemental data

Table S1: Monoclonal antibodies used in immunocytochemical characterization of glycopolymers in RET (mucilage and AC-DCs) and root (meristematic and elongation zones) under control and strong osmotic stress.

Glycopolymer	mAb	Epitope	Reference
Homogalacturonan	LM19	Homogalacturonan with low degree of esterification	[78]
	LM20	Homogalacturonan with high degree of esterification	
Rhamnogalacturonan I	LM5	[β(1-4)galactan]>3 Rhamnogalacturonan I	[79]
	LM6	[α(1-5) highly branched arabinan]5/6 Rhamnogalacturonan I	[80]
Xylogalacturonan	LM8	Xylogalacturonan associated with cell detachment and separation	[81]
Xyloglucan	LM25	Xylosylated/galactosylated xyloglucan (XXLG and XLLG)	[82]
Xylan	LM10	nonreducing end of xylans	[83]
Heteromanan	LM21	β-(1,4) linked mannan from DP2 to DP5 (mannan, glucomannan and	[84]
Arabinogalactan protein	JIM8	unknown	[85]
	JIM13	βGlcA-(1,3)-αGalA-(1,2)-Rha	[86,87]
	JIM14	internal epitope on β-1,6-linked galactans	[83]
	JIM16	β-1,3-linked galactan backbone when substituted with a single β-1,6-linked Gal	
Extensin	LM1	unknown	[88]
	JIM12	unknown	[89]

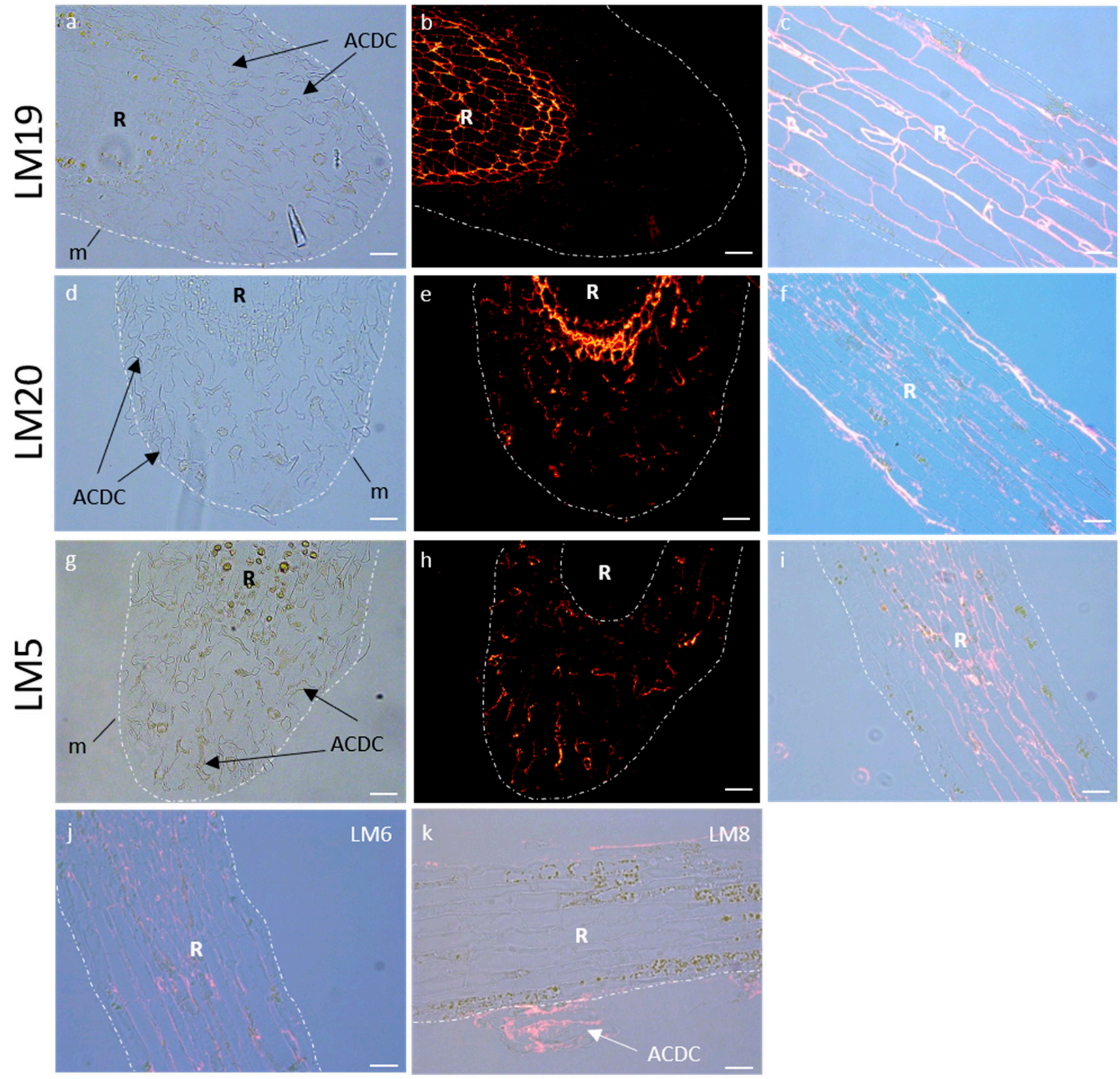

Figure S1. Immunocytochemical characterization of pectins in a control *P. nigra* root tip. LM19 weakly esterified homogalacturonan (a-c), LM20 highly esterified homogalacturonan (d-f), LM5 galactan chains from rhamnogalacturonan I (g-i), LM6 arabinan chains from rhamnogalacturonan I (j), LM8 xylogalacturonan (k). Left column: bright field images defining mucilage and AC-DC (except j), central column: corresponding images with fluorescence (except k), right column (and j, k): overlay of bright field/fluorescence images of elongation zone. RET labeling showed the detection of LM19 and LM5 in the AC-DC cell wall and no labeling of LM20. Root elongation zone presented a strong signal with LM19, contrary to LM20, LM5 and LM6 which showed a light signal. No signal was detected with LM8. AC-DC: root associated cap derived cell, m: mucilage, R: root. Bars: 25 μm.

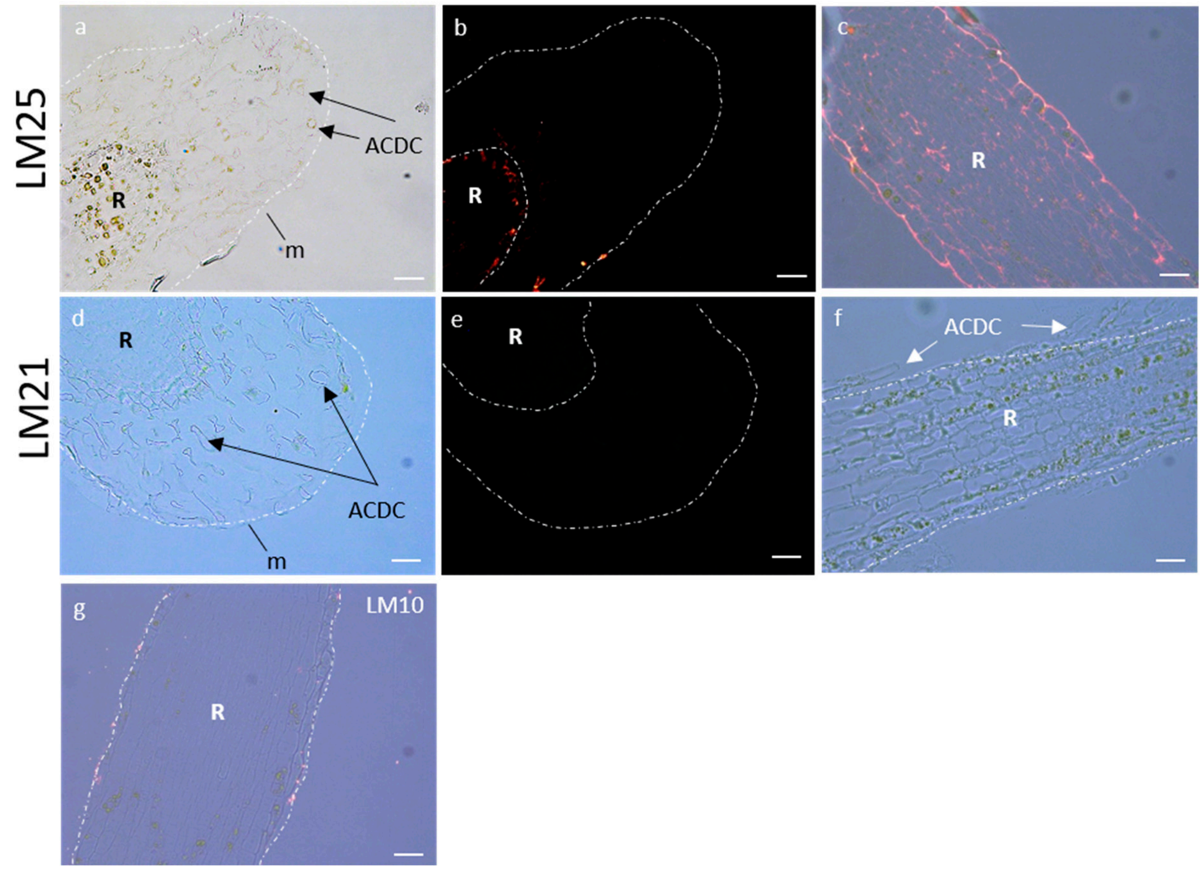

Figure S2. Immunocytochemical characterization of hemicelluloses in a control *P. nigra* root tip. LM25 galactosylated xyloglucan (a-c), LM21 heteromannan (d-f), LM10 xylan (g). Left column: bright field images defining mucilage and AC-DC (except g), central column: corresponding images with fluorescence, right column (and g): overlay of bright field/fluorescence images of elongation zone. RET labeling showed no detection of LM25 and LM21. Root elongation zone presented a light signal with LM25, contrary to LM21 and LM10 which showed no signal. AC-DC: root associated cap derived cell, m: mucilage, R: root. Bars: 25 μm.

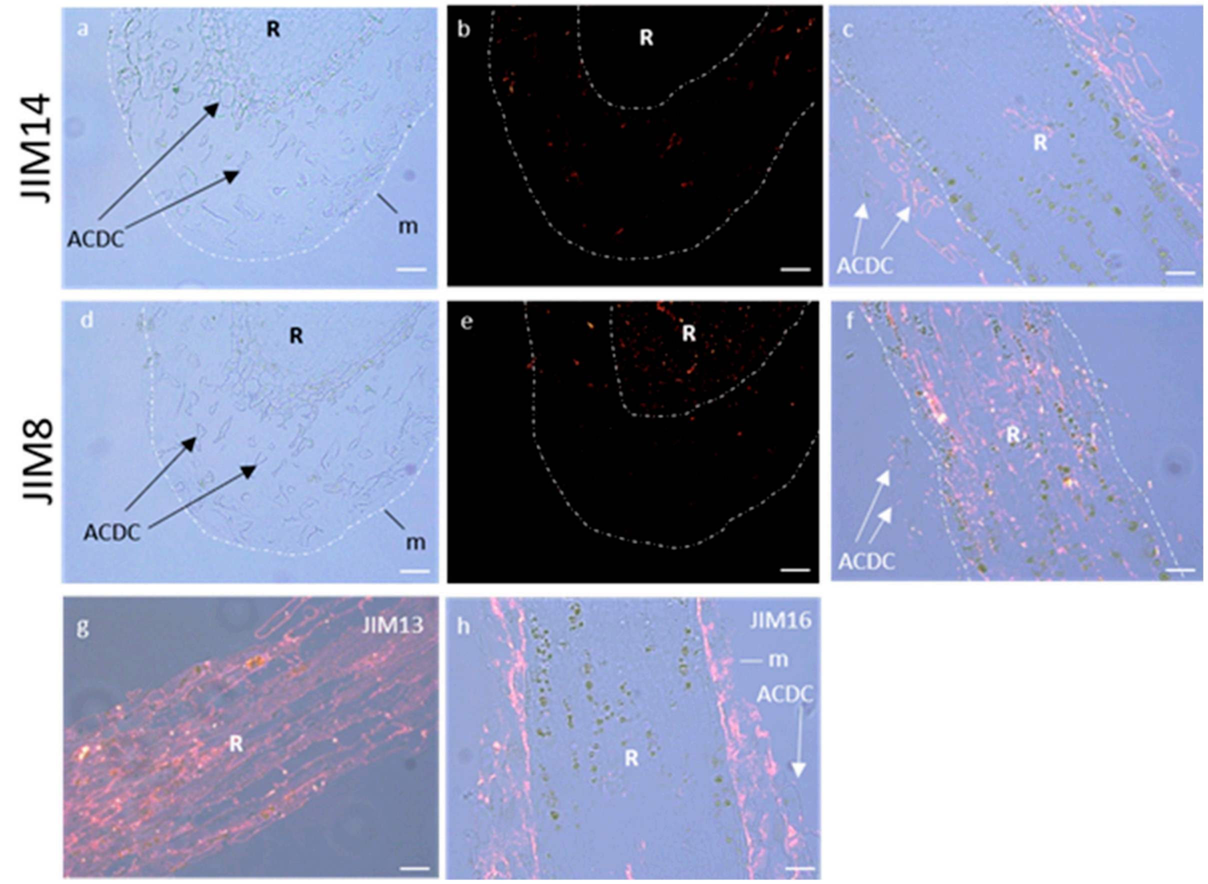

Figure S3. Immunocytochemical characterization of arabinogalactan proteins in a control *P. nigra* root tip. JIM14 (a-c), JIM8 (d-f), JIM13 (g), JIM16 (h). Left column: bright field images defining mucilage and AC-DC (except g), central column: corresponding images with fluorescence (except h), right column (and g, h): overlay of bright field/fluorescence images of elongation zone. RET labeling showed a weak fluorescence signal of JIM14 on the AC-DC cell wall and no signal with JIM8. Root elongation zone presented a strong signal with JIM8 and JIM13, contrary to JIM14 and JIM16 which showed no signal. AC-DC: root associated cap derived cell, m: mucilage, R: root. Bars: 25 μm.

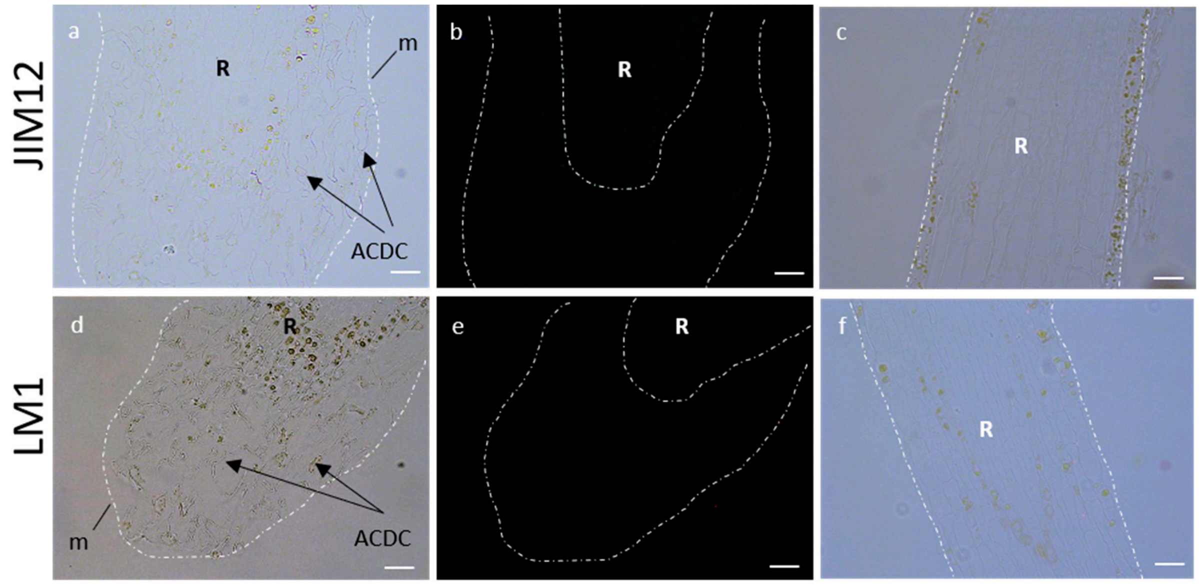

Figure S4. Immunocytochemical characterization of extensins in a control *P. nigra* root tip.JIM12 (a-c), LM1 (d-f). Left column: bright field images defining mucilage and AC-DC, central column: corresponding images with fluorescence, right column: overlay of bright field/fluorescence images of elongation zone. RET and root labeling showed no detection of JIM12 and LM1. AC-DC: root associated cap derived cell, m: mucilage, R: root. Bars: 25 μm.

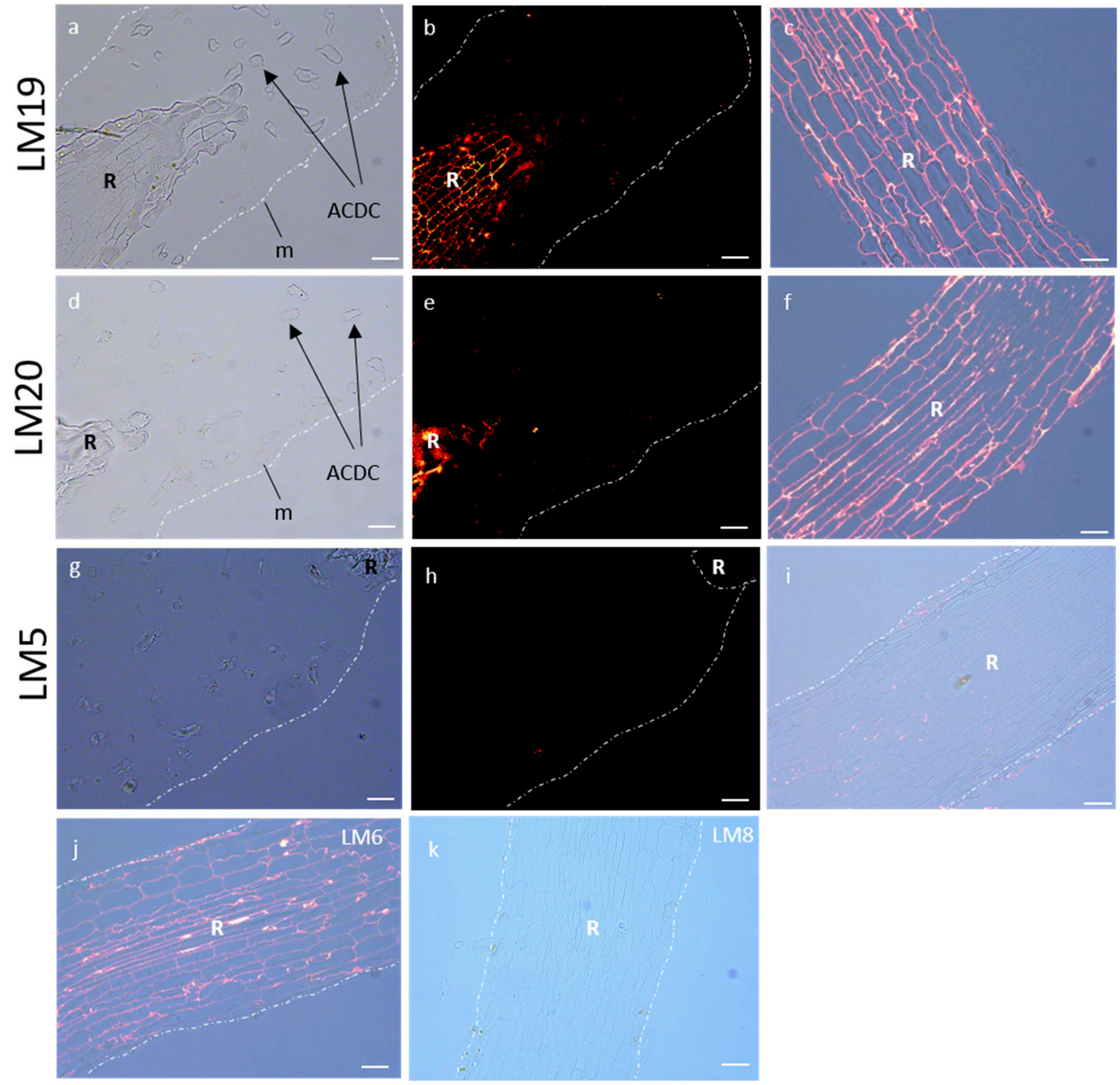

Figure S5. Immunocytochemical characterization of pectins in a *P. nigra* root tip under osmotic stress. LM19 weakly esterified homogalacturonan (a-c), LM20 highly esterified homogalacturonan (d-f), LM5 galactan chains from rhamnogalacturonan 1 (g-i), LM6 arabinan chains from rhamnogalacturonan 1 (j), LM8 xylogalacturonan (k). Left column: bright field images defining mucilage and AC-DC (except j), central column: corresponding images with fluorescence (except k), right column (and j, k): overlay of bright field/fluorescence images of elongation zone. RET labeling showed no detection of LM19, LM20 and LM5. Root elongation zone presented a strong signal with LM19, LM20 and LM6 contrary to LM5 which showed a light signal. No signal was detected with LM8. AC-DC: root associated cap derived cell, m: mucilage, R: root. Bars: 25 µm.

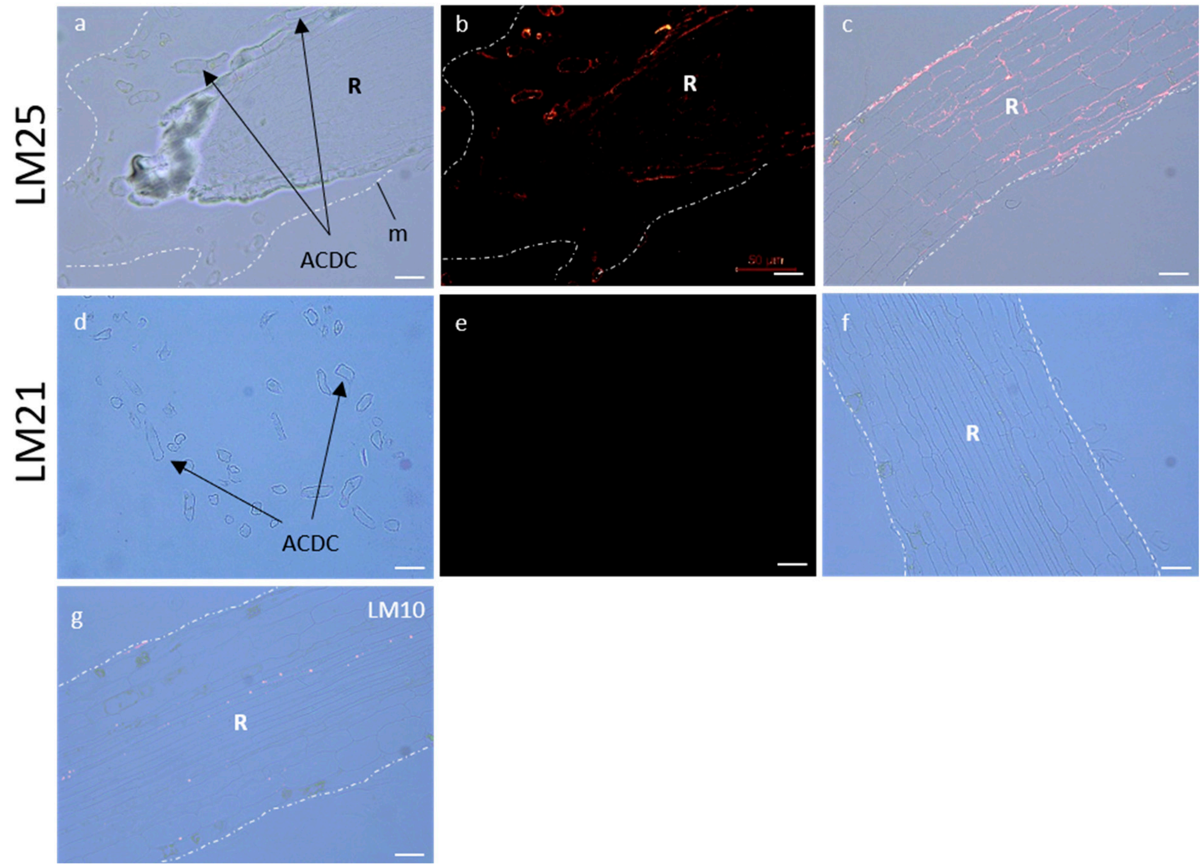

Figure S6. Immunocytochemical characterization of hemicelluloses in a *P. nigra* root tip under osmotic stress. LM25 galactosylated xyloglucan (a-c), LM21 heteromannan (d-f), LM10 xylan (g). Left column: bright field images defining mucilage and AC-DC (except g), central column: corresponding images with fluorescence, right column (and g): overlay of bright field/fluorescence images of elongation zone. RET labeling showed a weak fluorescence signal of AC-DC cell wall with LM25 and no signal with LM21. Root elongation zone presented a light signal with LM25, contrary to LM21 and LM10 which showed no signal. AC-DC: root associated cap derived cell, m: mucilage, R: root. Bars: 25 µm.

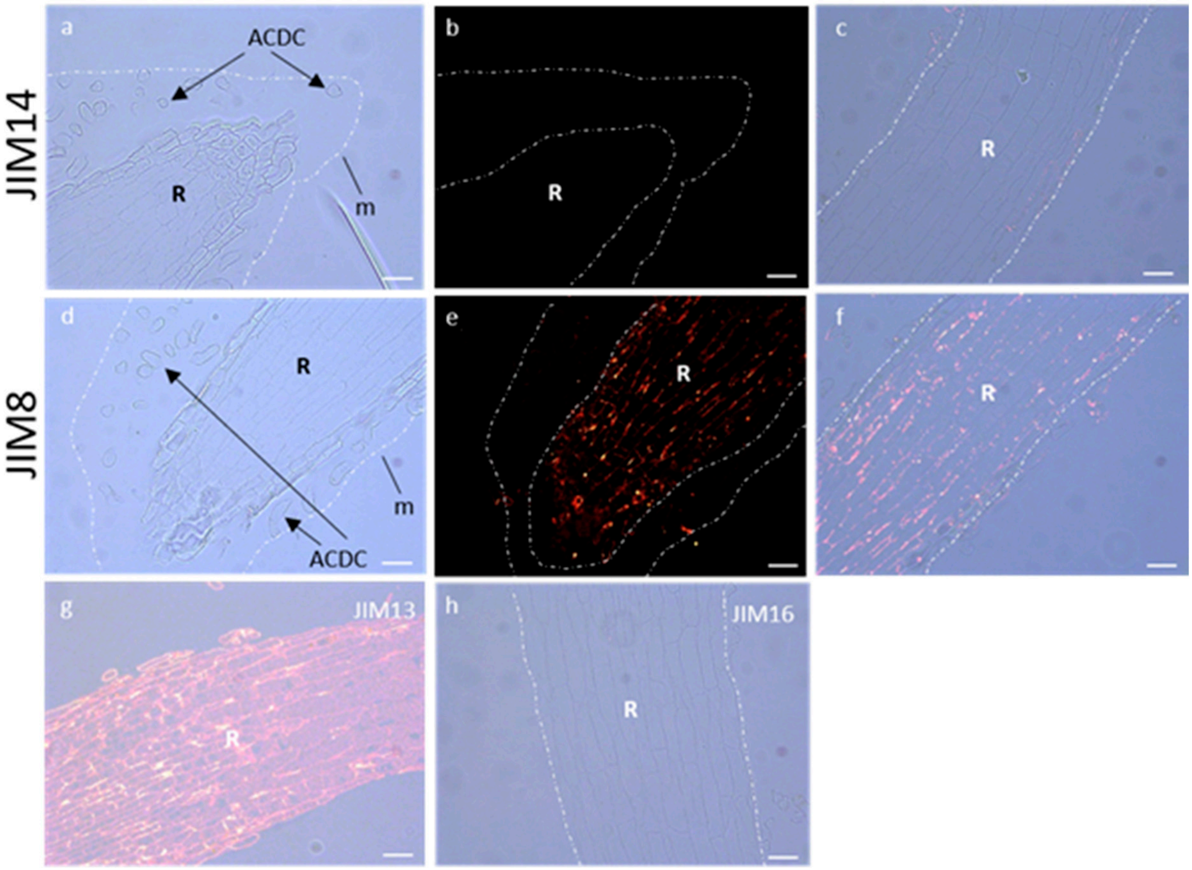

Figure S7. Immunocytochemical characterization of arabinogalactan proteins in a *P. nigra* root tip under osmotic stress. JIM14 (a-c), JIM8 (d-f), JIM13 (g), JIM16 (h). Left column: bright field images defining mucilage and AC-DC (except g), central column: corresponding images with fluorescence (except h), right column (and g, h): overlay of bright field/fluorescence images of elongation zone. RET labeling showed no fluorescence signal with JIM14 and JIM8. Root elongation zone presented a strong signal with JIM13 and JIM8, contrary to JIM14 and JIM16 which showed no signal. AC-DC: root associated cap derived cell, m: mucilage, R: root. Bars: 25 μm.

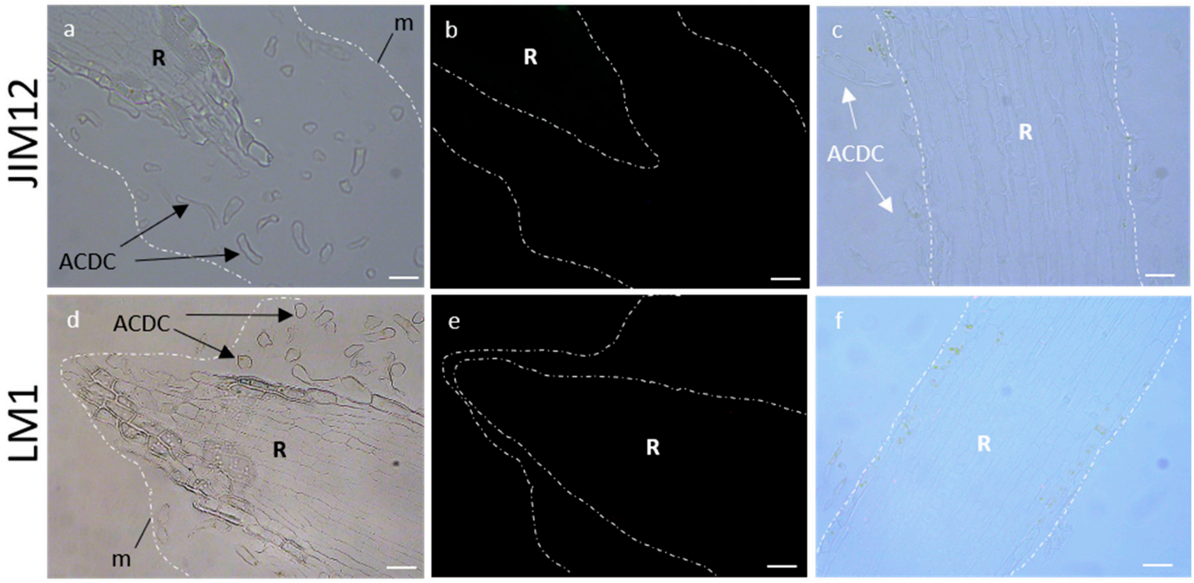

Figure S8. Immunocytochemical characterization of extensins in a *P. nigra* root tip under osmotic stress.JIM12 (a-c), LM1 (d-f). Left column: bright field images defining mucilage and AC-DC, central column: corresponding images with fluorescence, right column: overlay of bright field/fluorescence images of elongation zone. RET and root labeling showed no detection of JIM12 and LM1. AC-DC: root associated cap derived cell, m: mucilage, R: root. Bars: 25 μm.
